# Effect of short term aerobic exercise on fasting and postprandial lipoprotein subfractions in healthy sedentary men

**DOI:** 10.1186/s12944-015-0148-5

**Published:** 2015-11-25

**Authors:** Peter Sabaka, Peter Kruzliak, David Balaz, Andrea Komornikova, Denisa Celovska, Giovanni Cammarota, Katarina Kusendova, Matej Bendzala, Luis Rodrigo, Andrej Dukat, Taeg Kyu Kwon, Magdalena Chottova Dvorakova, Ludovit Gaspar

**Affiliations:** 2nd Department of Internal Medicine, Comenius University and University Hospital, Bratislava, Slovak Republic; 2nd Department of Internal Medicine, St. Anne’s University Hospital and Masaryk University, Pekarska 53, 656 91 Brno, Czech Republic; Division of Internal Medicine and Gastroenterology, Catholic University of Sacred Heart, A. Gemelli Medical School, Rome, Italy; Department of Gastroenterology, Central University Hospital of Asturias (HUCA), Oviedo, Spain; Department of Immunology, School of Medicine, Keimyung University, Dalseo-Gu, Daegu South Korea; Department of Physiology, Charles University in Prague, Faculty of Medicine in Pilsen, Pilsen, Czech Republic; Biomedical Centre, Charles University in Prague, Faculty of Medicine in Pilsen, Pilsen, Czech Republic

**Keywords:** Exercise, Lipoprotein profile, Lipoprotein subfractions, Postprandial lipoprotein profile

## Abstract

**Background:**

Our goal was to investigate the effect of short term exercise on fasting and postprandial lipoprotein profile.

**Methods:**

Healthy sedentary men exercised 20 min for four days. The intensity of exercise was modulated to maintain 75–80 % of a calculated HRmax. Before and after the exercise program, fasting and postprandial (4 h after standard meal) concentrations of lipoprotein subfractions were measured by an electrophoresis in polyacrylamide gel and total concentrations of TAG, LDL and HDL by enzymatic colorimetric method. After 2 days of rest, fasting and postprandial concentrations of lipoprotein fractions and subfractions were measured to determine a persistency of a changes in the lipoprotein profile.

**Results:**

4 days of physical exercise led to statistically significant decrease of concentration of triacylglycerol in fasting (76.29 ± 20.07, 53.92 ± 10.90, *p* < 0.05) and postprandial state (139.06 ± 23.72, 96.55 ± 25.21, *p* < 0.05) VLDL in fasting (21.88 ± 3.87, 18.00 ± 3.93, *p* < 0.05) and postprandial state (23.88 ± 3.52, 19.25 ± 3.62, *p* < 0.05), total cholesterol in fasting (162.26 ± 23.38, 148.91 ± 17.72, *p* < 0.05) and postprandial state (163.73 ± 23.02, 150.08 ± 18.11, *p* < 0.05). Atherogenic medium LDL decreased also in fasting (9.89 ± 3.27, 6.22 ± 2.55, *p* < 0.001) and postprandial state (8.88 ± 6.51, 6.88 ± 5.57, *p* < 0.001). However decrease of large IDL (25.38 ± 3.54, 23.88 ± 3.91, *p* < 0.05) and large LDL particles (42.89 ± 11.40, 38.67 ± 9.30) was observed only in postprandial state. Total HDL concentration remained unchanged but we observed statistically significant decrease of small HDL particles in fasting (6.11 ± 2.89, 4.22, *p* < 0.05) and postprandial state (6.44 ± 3.21, 4.56 ± 1.33, *p* < 0.05). Concentration of these particles are associated with progression of atherosclerosis. All changes of fasting and postprandial lipoprotein profile disappeared after 2 days of rest.

**Conclusion:**

Just 4 daily settings of 20 min of physical exercise can lead to significant positive changes of fasting and postprandial lipoprotein profile.

## Introduction

There is a large body of evidence to support the major role of regular physical activity in a cardiovascular prevention [1, 2]. Sedentary lifestyle and lack of regular exercise is associated with accumulation of other cardiovascular risk factors such as diabetes, abdominal obesity, dyslipidaemia and arterial hypertension [3]. Physiologic mechanism which mediates these positive effects is not fully understood yet. There is an evidence that physical exercise improves endothelial function [4] and also increases insulin sensitivity [5, 6]. Possibly improvement of lipoprotein profile plays significant role too [7–11]. Evidence of positive effects of physical activity on plasma lipoproteins comes mostly from the studies in which just fasting lipoprotein profile changes were observed [7–12]. However, lipoprotein metabolism is in fasting state only for few hours in the morning before the first meal [13]. Therefore, the postprandial lipoprotein profile may represent better the state of lipoprotein metabolism and may reflect better the lipoprotein cardiovascular risk factors. This theory is supported by the finding that non-fasting triacylglycerolaemia better predict cardiovascular events than fasting TAG concentration [13, 14]. Previous studies found decrease of postprandial TAG and VLDL concentration after short term exercise [15–18]. However, effects of short term aerobic exercise on all lipoprotein subfractions, especially in postprandial state, has not been described in detail yet. It is also unclear how fast the physical exercise may induce the significant changes of lipoprotein profile, because exercise training in the studies that measured complex changes of lipoprotein profile lasted usually more weeks [7–13, 19].

## Results

All volunteers completed the trial and maintained required intensity during all exercise settings and none of them were excluded due to inability to follow exercise protocol. The mean age of the volunteers was 27.2 ± 1.81 years, BMI 22.0 ± 1.47 kg/m^2^ and waist circumference 85.0 ± 5.20 cm. The mean power during exercises was 125.5 ± 17.23 W and exercise intensity 77.5 ± 1.434 % of HR max. Basal TAG, lipoprotein fractions and subfractions concentrations are shown in Tables 1, 2, 3 and 4. Fasting and postprandial concentrations of total cholesterol decreased significantly after exercise period, however after 2 days of rest the concentrations of cholesterol were not different from the their basal values (Table 1). The concentration of fasting and postprandial TAG levels aslo decreased significantly after 4 days of exercise (Table 1). However, there was no statistically significant difference in fasting or postprandial triacylglycerolaemia between the concentration before exercise and after 2 days of rest (Table 1).

**Table 1:** Changes of fasting and postprandial concentrations of cholesterol, TAG and VLDL after 4 days of training and then after 2 days of rest

	Baseline	4 days of training	2 days of rest	*P* (ANOVA)
Cholesterol (fasting)	162.26 ± 23.38	148.91 ± 17.72*	150.73 ± 23.86	<0.05
Cholesterol (postprandial)	163.73 ± 23.02	150.08 ± 18.11*	157.70 ± 18.74	<0.05
TAG (fasting)	76.29 ± 20.07	53.92 ± 10.90*	65.54 ± 19.28	<0.05
TAG (postprandial)	139.06 ± 23.72	96.55 ± 25.21*	138.44 ± 24.83	<0.05
VLDL (fasting)	21.88 ± 3.87	18.00 ± 3.93*	20.88 ± 4.88	<0.05
VLDL (postprandial)	23.88 ± 3.52	19.25 ± 3.62*	25.75 ± 3.85	<0.05

*TAG* triacylglycerol, *VLDL* very low density lipoprotein, *NS* non significant, *p < 0.05 – statistical significant difference from the baseline by post hoc test, Parameters presented as mean values ± standard deviation in mg/dl

**Table 2:** Changes of fasting and postprandial concentrations of large, medium and small IDL after 4 days of training and then after 2 days of rest

	Baseline	4 days of training	2 days of rest	*p* (ANOVA)
Large IDL (fasting)	26.44 ± 5.59	26.33 ± 5.98	25.67 ± 6.10	NS
Large IDL (postprandial)	25.38 ± 3.54	23.88 ± 3.91*	25.38 ± 5.18	<0.05
Medium IDL (fasting)	8.33 ± 2.87	9.44 ± 2.70	8.67 ± 2.35	NS
Medium IDL (postprandial)	6.44 ± 2.51	7.11 ± 2.52	8.00 ± 5.00	NS
Small IDL (fasting)	16.56 ± 4.39	17.56 ± 5.22	18.22 ± 3.99	NS
Small IDL (postprandial)	15.11 ± 2.26	13.33 ± 4.36	15.78 ± 3.42	NS

*IDL* intermediate density lipoprotein, *NS* non significant, *p < 0.05 – statistical significant difference from the baseline by post hoc test, Parameters presented as mean values ± standard deviation in mg/dl

**Table 3:** Changes of fasting and postprandial concentration of LDL and its subfractions (large, medium and small LDL) after 4 days of training and then after 2 days of rest

	Baseline	4 days of training	2 days of rest	*p* (ANOVA)
Total LDL (fasting)	114.46 ± 22.40	103.21 ± 17.00	104.99 ± 14.93	0.063
Total LDL (postprandial)	113.73 ± 17.80	103.40 ± 14.40*	106.50 ± 14.51	<0.05
Large LDL (fasting)	41.89 ± 11.81	39.44 ± 10.18	41.11 ± 12.35	NS
Large LDL (postprandial)	42.89 ± 11.40	38.67 ± 9.30*	37.56 ± 7.30	<0.05
Medium LDL (fasting)	9.89 ± 3.27	6.22 ± 2.55*	6.89 ± 2.36	<0.001
Medium LDL (postprandial)	8.88 ± 6.51	6.88 ± 5.57*	7.00 ± 2.73	<0.001
Small LDL (fasting)	0.44 ± 0.29	0.00 ± 0.00	0.33 ± 0.33	NS^a^
Small LDL (postprandial)	0.00 ± 0.00	0.00 ± 0.00	0.00 ± 0.00	NS^a^

^a^Results for small dense LDL (LDL 3–7) are not representative because of very small number of volunteers with detectable concentrations of these particles at the baseline

*LDL* low density lipoprotein, *NS* non significant, *p < 0.05 – statistical significant difference from the baseline by post hoc test, Parameters presented as mean values ± standard deviation (or standard error for parameters without normal distribution – medium LDL (fasting), small LDL (fasting and postprandial)) in mg/dl

Total LDL concentration is sum of concentrations of LDL subfractions and medium and small IDL (by Quantimetrics Lipoprint LDL system)

**Table 4:** Changes of fasting and postprandial concentration of HDL and its subfractions (large, medium and small HDL) after 4 days of training and then after 2 days of rest

	Baseline	4 days of training	2 days of rest	*p* (ANOVA)
Total HDL (fasting)	50.66 ± 10.52	49.65 ± 9.17	49.92 ± 10.63	NS
Total HDL (postprandial)	50.39 ± 10.13	48.76 ± 8.92	49.23 ± 9.21	NS
Large HDL (fasting)	15.50 ± 5.02	15.10 ± 4.51	14.80 ± 5.27	NS
Large HDL (postprandial)	16.56 ± 5.92	16.56 ± 5.53	16.22 ± 5.36	NS
Medium HDL (fasting)	23.70 ± 2.87	23.30 ± 2.41	25.00 ± 3.59	NS
Medium HDL (postprandial)	24.10 ± 3.18	22.80 ± 2.94*	23.70 ± 3.56	<0.05
Small HDL (fasting)	6.11 ± 2.89	4.22 ± 1.72*	5.00 ± 2.69	<0.05
Small HDL (postprandial)	6.44 ± 3.21	4.56 ± 1.33*	6.00 ± 2.06	<0.05

*HDL* high density lipoprotein, *NS* non significant, *p < 0.05 – statistical significant difference from the baseline by post hoc test, Parameters presented as mean values ± standard deviation in mg/dl

We obtained similar results for fasting and postprandial VLDL (Table 1). Fasting concentration of VLDL-remnants (large IDL particles) did not change, but postprandial concentration of VLDL-remnants decreased after 4 days of exercise, however, it was not significant (Table 2). This change disappeared after 2 days of rest and there was no significant difference between basal concentration and concentration after resting period (Table 2). We observed no statistically significant changes of mean concentrations of fasting or postprandial concentrations of medium or small IDL particles.

Although, we did not observed statistically significant decrease of total fasting LDL after the exercise period, we revealed significant decrease in postprandial LDL (Table 3). Fasting concentration of large LDL (LDL 1) did not decreased significantly but there was significant reduction in postprandial concentration of LDL 1 (Table 3). The atherogenic medium LDL (LDL 2) decreased significantly in fasting and postprandial state (Table 3). Only 2 of 10 volunteers had detectable levels of small dense LDL particles (LDL 3–7) in fasting state and none in the postprandial state, so we were unable to observe the changes of concentrations of these particles. All changes of LDL particle induced by exercise disappeared after 2 days of rest.

Total concentrations of fasting and postprandial HDL did not change significantly (Table 4), but we observed changes in some HDL subfractions. Fasting and postprandial small HDL and postprandial medium HDL particles decreased after exercise period (Table 4). Changes in HDL subfractions disappeared after 2 days of rest (Table 4).

## Discussion

In this study, we documented that the short term exercise induces various changes of fasting and postprandial concentration of lipoprotein subfractions in the population of healthy, sedentary men. These changes represent decrease of known atherogenic subfractions and therefore can be regarded as benefitial. Previously conducted studies observed some of these changes only in fasting state. However lipoprotein metabolism is in fasting state only few hours in the morning before first meal [13]. The postprandial lipoprotein profile, therefore, may better reflect the overall lipoprotein cardiovascular risk factors. This conclusion is supported by the evidence that postprandial triacylglycerolemia is better predictor of cardiovascular events than fasting triacylglycerolaemia [12–14]. Postprandial concentration of TAG and various lipoprotein subfractions significantly differs from their fasting concentrations [20]. Previous studies found decrease of postprandial TAG and VLDL after one setting of exercise [15–17]. These changes may influence other lipoproteins, since VLDL is precursor of IDL and is also involved in LDL and HDL metabolism [21]. We observed changes of postprandial concentrations of various lipoprotein subfractions and some of them has been found only during postprandial measurement (decrease of large IDL, large LDL, medium HDL). Another significant finding is that just 4 settings of 20 min lasting aerobic exercise lead to significant changes of lipoprotein profile by decreasing the atherogenic lipoprotein fractions and subfractions. That concludes that significant changes of lipoprotein metabolism induced by exercise starts much earlier than previously believed.

### TAG, VLDL and IDL

We observed decrease of fasting TAG and VLDL which is consistent with previous findings [6, 8, 10, 11]. Postprandial triacylglycerolaemia independently predicts cardiovascular events [13, 14]. Decrease of postprandial triaclyglycerolemia is probably caused by increase of lipolysis, since physical exercise up-regulates activity of muscle lipoprotein lipase (LPL) [6]. Exercise also inhibits VLDL secretion from the liver which is probably due to decrease of amount of available substrate [22]. We observed decrease of VLDL remnants (large IDL), but only in postprandial conditions. These particles probably possess significant atherogenic potential [23] so their reduction can be regarded as beneficial.

### Cholesterol, LDL and LDL subfractions

Fasting and postprandial concentrations of total cholesterol decreased after 4 days of exercise. Total LDL and concentration of large LDL, like the concentration of VLDL remnants significantly decreased only in postprandial state, however we found trend to decrease in fasting total LDL concentration (*p* = 0.063). Previous studies that found decrease of fasting LDL combined physical activity with calorie restriction as a part of complex weight loss programme [8, 24]. Study that compared effects of calorie restriction and exercise, found reduction of fasting LDL only in the cohort with calorie restriction [9]. Decrease of medium LDL particles (LDL 2) was found in our cohort in both fasting and postprandial state. This decrease was more pronounced compared to decrease of large LDL. This effect was not previously described, because previous studies did not measure medium LDL separately [8] or found no significant change in their concentration [9]. Concentration of medium LDL was associated with an increased risk of the premature myocardial infarction [25]. Substantial decrease of medium LDL might be associated with decrease of VLDL concentration. Cholesteryl ester transfer protein (CETP) mediates exchange of TAG and cholesterol ester between VLDL and LDL. In case of high VLDL concentration, LDL particles are enriched with triacylglycerol in interaction with VLDL particles. Triacylglycerol enriched LDL is substrate for hepatic lipase (HL) which catalyses the hydrolysis of its core and surface lipids leading to formation of smaller LDL particles [21]. Therefore, decrease of fasting and postprandial VLDL may lead to increase in mean LDL particle size. The same mechanism probably leads to decrease of small dense LDL previously observed after the training of longer duration [8, 9]. We were unable to observe the effect of exercise on small dense LDL (LDL 3–7) because concentration of these particles were below the limit of detection in vast majority of volunteers. That can be explained by selection of volunteers from the population of young, healthy and non-obese adults, because the presence of small dense LDL particles is associated with abdominal obesity, diabetes, hypertriacylglycerolaemia and older age [26, 27].

### HDL and HDL subfractions

Many prospective studies reported increase in total HDL cholesterol concentration under condition of regular physical training [7, 8, 10]. In our study, 20 min of aerobic exercise for 4 days were not sufficient to induce significant increase of total HDL. In contrary, we found decrease of postprandial medium HDL and fasting and postprandial small HDL particles. Concentration of small HDL particles are positively associated with risk of atherosclerosis progression and also correlates with other cardiovascular risk factors [28–35]. Small HDL as well as small dense LDL correlate with concentration of VLDL [28–35]. Therefore, decrease of fasting and postprandial VLDL may contribute to decrease small HDL. Dutheil et al. described similar changes of small and medium HDL after 20 days of complex physical training and diet program [8]. Our findings conclude that changes of HDL subfractions are present already after 4 days of aerobic exercise.

### Study limitations

To quantify the intensity of exercise we rather used estimated HR max calculated from age using equation by Tanaka et al. (208–0.7 × age) than measure exact VO2max by spiroergometry. Tanaka et al. concluded that HRmax is predicted in healthy subjects, to a large extent, by age alone and is independent of gender and habitual physical activity status. This conclusion is based on findings of large metaanalysis comparing laboratory obtained and calculated HRmax. Correlation between age and HRmax obtained from the metaanalysis is very high (*r* = − 0.90) which underlines an accuracy of the estimated HR max. [30]. We found the use of calculated HRmax fully acceptable, because we did not compare any interindividual differences in exercise intensity dependable variables and taking to account high correlation between estimated and measured values obtained by other studies [31].

Our study included 10 volunteers so sample size may appear as too low to detect smaller effect sizes. The number of volunteers is based on power analysis we performed using variances within groups and variances explained by effect from pilot study of effect of 4, 7 and 14 days of moderate exercise on concentration of plasma lipoprotein subfractions measured by linear electrophoresis in population of young healthy individuals [36]. We determined effect sizes for all variables that are expected to be affected by exercise according to previous studies by other authors [7–11]. For VLDL the interindividual variance within group, variance explained by effect and effect size was 48.7, 2.56, 0.229; for VLDL remnants 16, 0.9, 0.237; for large LDL 90.25, 1.26, 0.118, for medium LDL 39.7, 2.56, 0.254 and for HDL 72.25, 1.56, 0.136. Sample size of 10 volunteers appeared to be acceptable with power at least 0.90 for a detection of as low as 5 % change of means of variables. To obtain sufficient power with relatively small effect sizes was allowed by high correlation among repeated measurements (from 0.76 for VLDL to 9.1 for large LDL). This is caused mainly by low intraassay variability observed in previous studies [35–39]. Accuracy is comparable to nuclear magnetic resonance spectroscopy which represents a gold standard (CV approximately 4 % for Lipoprint and NMR). Quantimetrix Lipoprint LDL System is also approved by the U.S. Food and Drug Administration for use in human clinical medicine [40]. We reduced the effect of small sample size using analysis of variance for repeated measures that is more robust than the student *T*-test or Wilcoxon test. We were encouraged by a fact that other authors successfully conducted research studying the effects of exercise on lipoprotein particles with similar number of volunteers (for example *n* = 11 in paper by Wooten et al. J Appl Physiol. 2009) [38].

Diet may also influence postprandial lipemia [39]. Wen studding only effect of exercise, to get representable results, this influence should be eliminated or at least minimised. Diet of the volunteers during our study was not strictly standardised, because radical change of diet (from their usual diet to standardised diet) might influence their lipoprotein profile. However, we educated the volunteers not to change their standard eating habits and to dine meal with approximately the same calorie and fat content at the day before blood sampling.

We measured postprandial lipoprotein concentration just at one point 4 h after meal. Therefore we were unable to observe the effect of exercise on lipoprotein changes during all postprandial period. We chose time frame of 4 h because triacylglycerolaemia and VLDL concentration is the highest at this time [15]. There is no standardised and clinically accepted protocol for postprandial lipoproteins measurement. We preferred the use of homogenous yoghurt rather than complex meal in effort to maintain the same dose of nutrients in each portion. The use of complex meal might better imitate the common eating habit of the volunteers, but it also might limitate the exact dosing of the fat. The amount of the fat in test meal was chosen to be within the range of fat load used by previous studies [15–17].

## Conclusions

Only 4 settings of aerobic exercise (20 min each setting) were able to induce significant changes of fasting and postprandial lipoprotein profile. We observed statistically significant decrease of concentration of VLDL, medium LDL, medium and small HDL and postprandial concentration of VLDL-remnants and medium HDL. These lipoprotein particles are believed to be associated with progression of atherosclerosis and development of its complications. However, these changes diminish after 2 days of rest.

## Methods

### Participants

We recruited 10 healthy sedentary men aged from 21 to 30 years who signed an informed consent and volunteered on a basis of a public announcement. The exclusion criteria were smoking, alcohol or drug abuse, known history of diabetes mellitus, malignancy, chronic kidney disease and cardiovascular disease, inborn heart defects, use of hypolipidaemics, corticosteroids, or any chronic medication. The volunteers with fasting serum glucose concentration above 5.6 mmol/l, TSH concentration outside the reference range and glomerular filtration calculated by the CKD-EPI equation of less than 1.5 ml/s, resting arterial blood pressure above 139/89 mmHg were excluded. The sedentary lifestyle was defined as a regular high intensity physical activity (running, swimming, cycling) less than 2 times per week or sustained regular low intensity physical activity (walking) lasting at least 30 min less than 3 times per week. The volunteers filled a questionnaire about medical history. Subsequently, waist circumference, height and weight were measured and BMI was calculated as weight in kilograms divided by the square of height in meters.

### Oral lipid load test

Blood was sampled after an overnight fast (at least 12 h) by venepuncture from cubital vein using the Vacutainer closed system with serum clot activator to measure fasting lipoprotein concentrations. After blood sampling volunteers consumed test meal represented by 420 g of cream yoghurt containing 58.0 g of fat, 53.76 g of carbohydrates, 10.5 g of proteins (743.2 kcal, 3108 kJ). Volunteers were instructed to avoid consuming any food or sweetened drinks and remain at rest until next blood sampling. Second blood sample to measure postprandial lipoprotein concentrations was obtained after 4 h of consuming test meal. Volunteers were educated not to change their eating habits. At the day before each blood sampling, volunteers should dine meal with approximately the same calorie and fat content to minimise possible effects of meal with extremely low or high calorie content on postprandial testing. To do so, they were recommended to use National Nutrient Database for Standard Reference Release of United States Department of Agriculture [41].

### Exercise period

The exercise trial started the next day after the first measurement of fasting and postprandial lipoproteins. The maximal heart rate (HRmax) was calculated using an equation by Tananka et al. [30]. Volunteers exercised on a cycle ergometer (Ergometer 900, GE Medical Systems, General Electric Company, Schenectady, New York). The exercise intensity was modulated by adjusting power to maintain heart rate between 75–80 % of the calculated HRmax. The heart rate during the exercise was monitored using a Polar Sporttester PST (Polar Electro, Kempele, Finland). The volunteers exercised in four following days, each day for 20 min. One day after termination of physical exercise volunteers underwent blood sampling in fasting state and the same protocol with lipid load test was performed. Volunteers were educated not to change eating habits.

### Resting period

After completing the exercise phase, volunteers remained at rest for 2 days. They were instructed not to perform a sustained low intensity physical activity for more than 15 min and to avoid high intensity physical activity. Fasting and postprandial lipoprotein concentrations were measured the following day after 2 day rest using the same protocol as before.

### Sample processing and analysis

After the blood sampling, the samples remained at room temperature for 30 min and subsequently a supernatant was separated by centrifugation for 10 min at 3,000 rpm. Some part of the supernatant was used for the determination of serum concentration of glucose, creatinine, TSH, total cholesterol, LDL, HDL and TAG. The second part of the supernatant was frozen at the temperature of −80 °C for analysis of lipoprotein classes and subfractions.

### Biochemical analyses

Total cholesterol, LDL, HDL, and TAG concentrations were determined by an enzymatic colorimetric method (Cobas Mira Plus, Roche Diagnostics GmbH, Montclair, NJ, USA). The concentrations of VLDL, IDL, LDL and HDL fractions and subfractions were determined by the linear electrophoresis in polyacrylamide gel (Quantimetrix Lipoprint LDL System and Quantimetrix Lipoprint HDL System, Rendo Beach, USA). Both methods use electrophoresis in tubes filled with 3 % polyacrylamide gel. 25 μl of serum was mixed with a 200 μl of a solution of the liquid gel and Sudan black, and added to the gel tube. Polymerization of the gel followed at room temperature for 30 min. Electrophoresis was performed for 1 h (3 mA/1 tube) and it was followed by densitometric reading and conversion to concentrations of lipoprotein fractions and subfractions using the Lipoware software (Quantimetrix, Rendo Beach USA). The Quantimetrix Lipoprint LDL System divides lipoproteins based on electrophoretic mobility and determinates the concentration of VLDL, large, medium and small IDL particles (large IDL particles corresponds with VLDL-remnants), large LDL particles (LDL 1), medium LDL particles (LDL 2), and small dense LDL particles (LDL3-7).

The Quantimetrix Lipoprint HDL System method determines 10 HDL subfractions divided into large, medium and small HDL.

### Statistical methods

The descriptive data were provided as a mean values ± standard deviation of average or standard error (for parameters without normal distribution). The D’Agostino-Pearson test and Kolmogorov-Smirnov test were used to verify the normal distribution of parameters in the cohorts. The normally distributed parameters were considered to be those which have passed the verification in both tests. The means of normal distributed parameters were compared by using one-way analysis of variance with repeated measures (ANOVA), followed by post hoc Tukey test to identify where the differences occurred. The means of parameters without normal distribution were compared by using Friedman test followed by Dunn’s multiple comparison test to identify where the differences occurred. A difference with the p value of less than 0.05 was considered as statistically significant. Before starting recruitment we performed power analysis using variances of each variables and correlation among repeated measures obtained by our previous pilot study using the lipoprotein linear electrophoresis method [36]. We determined effect sizes for all variables that are expected to be affected by exercise according to previous studies by other authors [7–11]. Sample size of 10 volunteers appeared to be acceptable with power at least 0.90 for each variable. Power analysis was conducted using G*Power 3.1.9.2 for Windows using algorithm for ANOVA with repeated measurements and within factors.

### Statement of human and animal rights

All procedures followed were in accordance with the ethical standards of the responsible committee on human experimentation (institutional and national) and with the Helsinki Declaration of 1975, as revised in 2008.

### Statement of informed consent

Informed consent was obtained from all patients for being included in the study.

## Abbreviations

CETP: Cholesterylester transfer protein
HDL: High-density lipoprotein
HL: Hepatic lipase
IDL: Intermediate-density lipoprotein
LDL: Low density lipoprotein
CKD-EPI: Chronic Kidney Disease Epidemiology Collaboration
TAG: Triacylglycerol
TSH: Thyroid stimulating hormone
VLDL: Very-low density lipoprotein
